# Qualitative Exploration of a Tele-exercise Program to Inform the Design of Adaptive Intervention Strategies for Adults With Multiple Sclerosis

**DOI:** 10.1016/j.arrct.2024.100423

**Published:** 2025-01-19

**Authors:** Yumi Kim, Elizabeth A. Barstow, Byron Lai, Dorothy W. Pekmezi, Hui-Ju Young, Jereme Wilroy, Soumya J. Niranjan, James H. Rimmer, Tapan Mehta

**Affiliations:** a Department of Physical Medicine and Rehabilitation, Heersink School of Medicine, University of Alabama at Birmingham, Birmingham, AL; b University of Alabama at Birmingham Research Collaborative, School of Health Professions, University of Alabama at Birmingham, Birmingham, AL; c Department of Occupational Therapy, School of Health Professions, University of Alabama at Birmingham, Birmingham, AL; d Division of Pediatric Rehabilitation Medicine, Heersink School of Medicine, University of Alabama at Birmingham, Birmingham, AL; e Department of Health Behavior, School of Public Health, University of Alabama at Birmingham, Birmingham, AL; f Department of Health Services Administration, School of Health Professions, University of Alabama at Birmingham, Birmingham, AL; g Department of Family and Community Medicine, Heersink School of Medicine, University of Alabama at Birmingham, Birmingham, AL

**Keywords:** Exercise therapy, Health behavior, Multiple sclerosis, Qualitative research, Rehabilitation

## Abstract

**Objective:**

(1) To examine the experiences and perceptions of people with multiple sclerosis (MS) who previously completed a 3-month tele-exercise program; (2) to identify modifiable factors regarding the program components and implementation procedures that could be used to develop an adaptive tele-exercise intervention.

**Design:**

A qualitative study using a semistructured interview guide.

**Setting:**

An extension of a comparative effectiveness trial delivered for 761 people with MS between 2016 and 2021. Interviews were conducted over Zoom or phone.

**Participants:**

Twenty-two adults with MS who completed the 3-month program (age range, 24-67y, 86% women, 73% Caucasian, 68% relapsing-remitting MS). Participants presented a wide range of mobility disabilities, ranging from no mobility limitation to wheelchair or scooter (n=22).

**Interventions:**

Not applicable.

**Main Outcome Measures:**

Thematic analysis of the transcribed interview led to the identification of desirable components and delivery mechanisms of the tele-exercise program.

**Results:**

Our analysis indicated the importance of individualized and ongoing modification of exercise program content to accommodate changes in participants’ functional abilities and health status. Participants reported an ideal timepoint of human support, preferably every 3 weeks, via phone or videoconference calls to capture the intervention modification needs (eg, exercise position, intensity, and clarification of exercise instruction). We further identified desirable components for behavioral modifications, such as inclusion of an exercise companion and self-monitoring tool and postintervention resources for sustaining exercise participation during follow-up periods.

**Conclusions:**

The findings of this study offer insights into tailoring future adaptive tele-exercise intervention designs aimed at promoting engaging and sustainable exercise participation in people with MS.

## Highlights

• Exercise programs should adapt to functional and health status changes over time.
• Healthcare providers should tailor support to participants' goals and needs.
• Sustaining exercise behavior in people with MS requires post-study resources and strategies.

Multiple sclerosis (MS) is a progressive neurodegenerative disease^1^ causing heterogeneous impairments in mobility, cognition, and symptoms, like fatigue and pain,^2^ requiring long-term rehabilitation. Despite the health benefits of regular physical activity (PA), including exercise training,^3^, ^4^, ^5^ people with MS engage in significantly lower levels of PA than the general population and adults with other health conditions.^6^, ^7^, ^8^

In response, researchers sought to address the persistent problem of physical inactivity in people with MS through exercise training or behavioral interventions.^3^ Tele-exercise^9^ further offers cost-efficient options that overcome transportation barriers.^10^ However, targeted, tailored exercise programs are needed as responses vary greatly among people with MS.^11^ Although some individuals experience large increase in PA after an intervention, others may demonstrate no change or even a decline.^12^, ^13^, ^14^ These variations may be influenced by factors such as disease severity and exercise barriers.^12^, ^13^, ^14^, ^15^, ^16^

Qualitative studies have suggested that people with MS want exercise programs with meaningful benefits and various ways that they can do the exercise.^17^, ^18^, ^19^ However, challenges with tele-exercise include technology issues, limited social support, and the need for continuous program modifications.^20^, ^21^, ^22^ These insights can guide tailored tele-exercise programs, but further feedback from participants is needed, especially to refine program for time-sensitive modifications to apply adaptive intervention or the Sequential, Multiple Assignment, Randomized Trial designs.^23^

This study sought to qualitatively explore the experiences and perceptions of participants with MS who completed a 3-month tele-exercise program to identify program components and implementation processes that are required for strong engagement. This study was part of the larger Tele-Exercise and Multiple Sclerosis (TEAMS) study, a cluster randomized controlled effectiveness trial involving 761 people with MS.^21^^,^^24^^,^^25^ The TEAMS study compared a 3-month tele-exercise intervention to the same intervention delivered in-clinic by a physical therapist followed by a 9-month follow-up. The original intervention, grounded in social cognitive theory,^3^^,^^26^^,^^27^ included educational articles aligned with the key constructs (eg, self-efficacy, outcome expectation, and self-regulatory strategies) and weekly automated interactive voice response (IVR) phone calls for feedback, encouragement, and support.

To better understand response heterogeneity in people with MS, we examined 2 groups: *responders* (clinically meaningful PA increases postintervention) and *low-responders* (minimal/no changes or decreases). This study sought to answer the following central research question: What are the participant's experiences and perceptions of the 3-month tele-exercise program, focusing on the differences between responders and low-responders? Unlike the previous program evaluation,^21^ which included both tele-exercise and in-clinic participants after the initial 3-month intervention period, this study specifically targeted tele-exercise participants who completed the full 1-year intervention and received videos offering 6 levels of mobility adaptations tailored based on a clinician-led Timed 25-Foot Walk Test. Participants previously interviewed in the program evaluation were excluded.

## Methods

The study was approved by a University Institutional Review Board and followed the Consolidated Criteria for Reporting Qualitative Research guidelines (Appendix I).^28^ Written informed consent was obtained from all participants.

### Philosophical assumptions and design

A narrative inquiry was chosen in this qualitative study underpinned by an interpretivism paradigm. We focused on the storied experiences of individuals (ie, narrators), which are based on their lives within the social worlds and particular perspectives on exercise participation with MS and lay out the meaning of those experiences in chronological order.^29^ Under an interpretivism approach, the research team employed 2 philosophical assumptions: ontological relativism^30^ and epistemological constructivism.^31^ The study design was led by multiple principal investigators, who are included as authors of this study, in collaboration with community stakeholders living with MS. Reflexivity statements of the research team are presented in text box 1.

Text Box 1 **The research team's reflexivity statements**

**Table:** 

The first analyst (Y.K.) was a PhD candidate in Rehabilitation Science. She had prior experience developing and implementing adapted exercise programs for individuals with various physical disabilities and chronic health conditions, including MS, within the field of Adapted Physical Activity. The second analyst (B.L.) was a postdoctoral fellow in the Department of Pediatric Division Rehabilitation, focusing his research on developing and implementing tele-exercise interventions for adults and children with disabilities. The critical friend in this study (E.A.B.) is prolific in the field of rehabilitation research, with qualitative expertise, particularly in understanding the occupational limitations and needs of individuals with vision impairments and related conditions as they relate to health and wellness. The first and second analysts are from a nonwhite group, whereas the critical friend is from a white group. All are currently engaged in activities aimed at enhancing the health-related quality of life for people with various disabilities.

Alt-text: Unlabelled box

### Sampling procedure and participants

**Fig 1:**
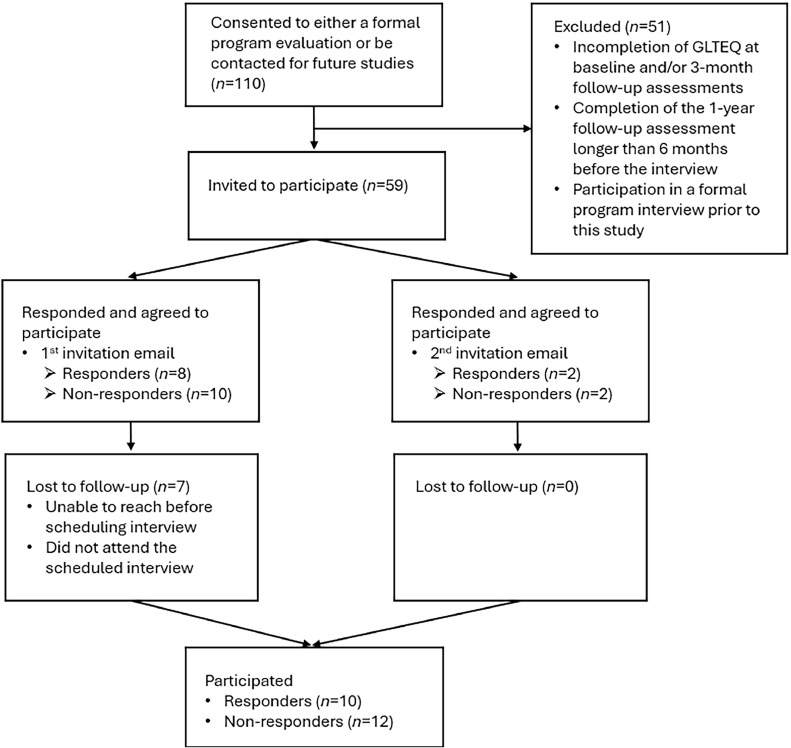
Participant flow.

Participants were recruited using purposeful sampling, including convenience and criterion-based methods, until reaching theoretical saturation (ie, until no relevant codes were generated from last 3 participants). The participants were a convenience sample recruited from the tele-exercise arm of the TEAMS study.^24^ Individuals, who had expressed interest in completing a postintervention interview or being contacted for future studies, were eligible if they completed the (1) 3-month intervention, (2) PA questionnaire for both baseline and 3-month follow-up assessments, and (3) the 1-year follow-up assessment no later than 6 months before the interview (November 2020-February 2021). Participant flow is shown in figure 1.

The criterion-based strategy sought individuals with diverse knowledge and experiences related to the phenomenon of interest to capture detailed, in-depth, and rich information.^32^ We specifically targeted 2 participant groups, *responders* and *low-responders*, based on their change in moderate to vigorous PA (MVPA) after the intervention. PA was measured using the Godin Leisure-Time Exercise Questionnaire (GLTEQ), which is a validated, self-report measure of PA in people with MS.^33^ A meaningful increase in MVPA was defined as a change greater than the 75th percentile of the entire sample distribution (n=377) after the 3-month tele-exercise intervention. Therefore, participants with a change in MVPA above this threshold (≥10 points on the GLTEQ) were classified as *responders*, whereas participants reporting minimal to no change or a decrease (<10 points on the GLTEQ) were classified as *low-responders*.

### Data collection

Data were collected as audio files by the first author using semistructured one-on-one interviews via telephone or Zoom conference call based on each participant's preference. The interviews were conducted using preplanned questions (table 1), which were framed around the key social cognitive theory constructs and further refined based on previous literature and our experiences in the field of MS research. Each interview lasted up to 1 hour and was audio-recorded. The recordings were then transcribed *verbatim* by an external transcription service*.* The accuracy of the transcribed audio files was checked by the primary analyst (Y.K.).

**Table 1:** Interview questions guides

1. What made you to join/sign up in this program?
2. At the start of the program, what were you expecting to get from the program?
2a. Did this change up to the mid of the program?
2b. Did this change from the mid to the end of the program?
3. (Considering this program was 100% online, no personal coaching or interaction) At the start of the program, how confident were you that you could do, stick to, or maintain the program?
3a. Did this change in the middle of the program? If yes, how different? If no, why not?
3b. Did this change after the program? If yes, how different? If no, why not?
4. Tell me about your overall perceptions of the program you participated in.
4a. Can you describe the positive experiences that you have had from the program?
4b. Can you describe the negative experiences that you have had from the program?
I would love to talk about the detailed program components. What you liked and disliked, how would you like to change.
5. Tell me about how you felt about the *exercise videos/regimen* at the start of the program.
5a. Did this change in the middle of the program? If yes, how different? If no, why not?
5b. Did this change after the program? If yes, how different? If no, why not?
6. Tell me about what factors (some things) that prevented you from the exercise. (barriers)
7. Tell me about what factors (some things) that helped you with the exercise. (facilitators)
8. What would you like to add or how would you like to change this program and its delivery?
9. Tell me about how you felt about the *newsletters*?
9a. Did this change in the middle of the program? If yes, how different? If no, why not?
9b. Did this change after the program? If yes, how different? If no, why not?
9c. Were you able to apply what you learned from the *newsletters* to your activities of daily living? Why/Why not?
10. Tell me about how you felt about the *automated calls (ie, IVR)* to monitor your progress and keep up with the program? Why/Why not?
10a. Did this change in the middle of the program? If yes, how different? If no, why not?
10b. Did this change after the program? If yes, how different? If no, why not?
11. Tell me how you felt about the technology that was used to deliver the program (app, tablet).
12. Tell me about your exercise or activity routine.
12a. Do you think your physical activity level has changed (more active vs less active vs same) compared with before?
13. Do you have anything else that you would like to add?

### Data analysis

Thematic analysis was used to identify patterns and summarize key features.^34^ Two analysts (Y.K., B.L.) performed independent, segment-by-segment coding for the *responder* and *low-responder* groups. *Initial codes* were identified by looking for common threads using specific words or phrases within passages and organized them into 5 categories: barriers, facilitators, likes, dislikes, and suggestions. The analysts then grouped these codes into logical categories (*themes*), categorizing into *2 time-periods*: (1) during the 3-month intervention and (2) after the formal intervention. Finally, *a list of modifications* was created, noting the needs and recommendations described by participants, providing further context for designing adaptive tele-exercise interventions for people with MS.

### Ensuring rigor

To maintain the transparency and trustworthiness of this study, 2 analysts performed independent, segment-by-segment coding and then discussed the themes and final coding scheme jointly. We also employed the “critical friend” approach to ensure that the research processes were appropriately conducted according to the chosen philosophical assumptions and to generate the relevance of the identified themes and subthemes.^30^ Accordingly, the analysts scrutinized all study matters (eg, study procedures, findings, and theoretical preferences themselves) as a reflexive method to enhance quality and transparency,^35^ particularly with explanations and interpretations of the data.

## Findings

Participant characteristics are summarized in table 2. Age, sex, MS type, and resident location were similar between groups, but differences were observed in race and disease severity. The low-responder group had fewer African Americans (17% vs 40%) and a higher proportion of participants with more severe MS (≥3 on the Patient Determined Disease Step^36^; 58% vs 30%) than the responder group.

**Table 2:** Participant characteristics (N=22)

ID	Age	Sex	Race	Location	MS Type	PDDS	Change in HCS
1	31	F	AA	Metro	RR	1	60
6	46	F	AA	Metro	RR	0	10
8	41	F	C	Metro	Unknown	1	15
12	24	F	C	Metro	Unknown	2	15
13	46	F	C	Micro	RR	3	10
14	25	F	C	Metro	Unknown	3	19
15	51	M	C	Micro	RR	6	30
19	55	F	AA	Metro	RR	2	10
20	67	F	C	Metro	RR	0	15
23	41	F	C	Micro	RR	1	39
Responders (n=10)	43 (13; 24-67)	9 F / 1 M	6 C/4 AA	7 Metro/3 micro	7 RR/3 unknown	2 (1-3)	
3	58	F	C	Metro	RR	4	4
4	49	F	C	Metro	RR	4	0
5	33	F	C	Micro	Progressive	3	−10
7	61	F	C	Metro	RR	1	4
9	41	F	C	Micro	RR	3	5
10	56	F	C	Metro	Unknown	4	0
11	53	M	C	Metro	Unknown	4	0
16	26	F	AA	Small Town	RR	0	−10
17	34	F	AA	Metro	RR	1	5
18	60	F	C	Metro	RR	1	−5
21	48	M	C	Metro	Progressive	7	−20
22	58	F	C	Metro	RR	2	−15
Nonresponders (n=12)	48 (12; 26-60)	10F/2M	10 C/2 AA	9 metro/2 micro/1 small town	8 RR/2 progressive/2 unknown	3 (1-4)	

NOTE. Age was reported as mean (standard deviation; range), and disease severity (ie, PDDS) was reported as median (IQR).

Abbreviations: AA, African American; C, Caucasian; F, female; HCS, health contribution score; M, male; Metro, metropolitan; Micro, micropolitan; PDDS, Patient Determined Disease Steps; RR, relapsing-remitting.

Four themes emerged related to the program components and delivery during the 3-month intervention period (Themes 1-4), and 1 theme related to study participation during the 9-month follow-up period (Themes 5). Key illustrative quotes and related descriptions are presented below, with additional quotes provided in table 3. A list of proposed modifications is presented in table 4. Although the data from the 2 groups were analyzed separately, the findings are presented together because of the similarities in responses.

**Table 3:** Themes, subthemes, and additional representative quotes

Theme 1. Need to meet exercise expectations with abilities	
(a) Nonchallenging exercises caused boredom or lack of interest.	Participant 8 remarked on previous experiences with Pilates and commented, “I thought it [*the program*] would be something that was going to make me push myself a little harder. My normal exercises were Pilates so I was used to doing that, that's why I said I was expecting something just a little bit more.” [Responder]
(b) Overly challenging exercises created feelings of frustration, anxiety, and loss of confidence.	Participant 13 commented on the challenges with balance and floor activities, “I just wanted to see what it would do and help me. Some things during it I could not do. Some of the exercises I just cannot do because A, my balance, getting, for instance, up and down on the floor—I can get down on the floor real easy but getting up is another question. I just fall. Yeah, there's things I just cannot do.” [Responder]
Theme 2. Preference for personalized content and tools to support exercise behavior change	
(a) General, irrelevant content created feelings of disinterest and disconnection.	Participant 11 commented on the educational articles that did not affect any behavior change. “I read them, but as far as did they make me exercise more or exercise less? No, they didn't have a bearing that way. I can't say that I really drew anything off of them that really made a difference.” [Low-responder]
(b) Lack of awareness of content.	Participant 1 noted that the main focus was on exercise videos. “I didn't even know that [articles]. That's news to me. … Like I literally would turn it on, go to my TEAM MS study and I would just stick to that, you know, whatever video I was on.” [Responder]
(c) Multiple communication channels to support exercise behavior modification.	Participant 21 reported about an exercise sheet as a self-management tool to track exercise performance and progress. “I just listed all of them [*the exercise movements*] and I grade out the boxes—you know, I kept it going per week. … I could put the checkmarks in and I knew which ones I was doing—which ones I had done, you know, to keep track of it for myself. And it was pretty helpful. If you all make it—well, it could be an Excel spreadsheet but if you make it, I believe, like a fillable PDF, something to that effect, then, the person can just mark each exercise without having to worry about accidentally manipulating the spreadsheet or anything like that. They just pop a checkmark in the box as they do the exercise. And that's pretty helpful.” [Low-responder]
Theme 3. Importance of accountability and human interaction to increase participation	
(a) Human connection enhances feelings of support.	Participant 8 suggested adding interactive options to enhance connection and support. “For me personally, I would probably say a check-in call maybe once every two to three weeks. And the reason why I just said that is because of to hear a person's voice is more, like I said the encouragement.” [Responder]
(b) Exercise companionship enhances motivation.	Participant 20 commented that exercise companionship can positively influence adherence and effort during exercise. “I found that to be helpful when I was doing some other kind of workouts and that way you don't want to let your buddy down. And also you're competitive against your buddy. And it would be somebody at your level. … And you're not just feel like you're out there.” [Responder]
Theme 4. Perceived barriers to program participation	
(a) Daily variation of MS symptoms.	Participant 5 commented on physical symptoms preventing participation and the need to manage conditions carefully to avoid exacerbation. “When I was feeling really fatigued or dealing with spasticity, I just couldn't get through the exercises—it was like my body was fighting against me. Some days were better, but on the hard days, I had to stop completely to avoid making it worse.” [Low-responder]
(b) Life stressors and responsibilities.	Participant 19 noted the challenge of balancing multiple responsibilities and the tendency to deprioritize exercise. “Between work, taking care of the kids, and just trying to keep the house in order, there's hardly any time left for myself. Exercise is always the first thing to go when the day gets too busy.” [Responder]
(c) Unexpected injury or health issues.	Participant 20 noted that unexpected health issues disrupted participation in the intervention. “I had an MS relapse halfway through, and it completely threw me off. Then there was the rotator cuff injury that made it nearly impossible to keep up with the exercises.” [Responder]
Theme 5. Resources to reengage participants in continued exercise	
(a) Perceived benefits motivate to continue the exercise.	Participant 13 commented on how observable improvements drive adherence. “After a while, I noticed that my sleep improved, and my overall body felt less tense. The exercises seemed to help with managing my MS symptoms, including pain.” [Responder]
(b) Postintervention resources as a reminder.	Participant 20 highlighted the importance of keeping motivation by offering dynamic and engaging program elements. “I think it would be good to have some variations or maybe just a new way to do things in the middle [*of the exercise videos*] after you've watched all the videos and you know what you're doing. You need something a little bit different to keep everything fresh and to keep people motivated.” [Responder]

**Table 4:** Modifications for future tele-exercise interventions for people with MS

Needs	Recommendations
Appropriate level of challenge over time to optimize their exercise participation (Theme 1 and 4)	Occasional human interaction with staff through phone or conference calls (every 3wk) to assess changes in needs and difficulties and make ongoing modifications (Theme 3)
	Exercise companionship based on personal preferences (eg, synchronous, expert-led group, or individual training sessions; asynchronous with no direct human involvement; hybrid from synchronous coaching to asynchronous, self-directed training) (Theme 3)
Optimization of exercise behavior chance (Theme 2)	Multiple ways of reporting and monitoring achievement (eg, attendance, exercise minutes), such as web application, email, or call/text back to automated system (Theme 2)
	Ask personal interests and knowledge level about MS, exercise, and coping mechanism during screening. Then, provide in-depth, relevant educational content and feedback (eg, MS-related symptoms, exercise history, and motivational factors/ways to deal with setbacks). (Theme 2)
Postintervention resources may reengage participants in exercise (Theme 5)	Summary video or paper copy of exercise materials without instruction (Theme 5)
	Additional set of exercise materials to continue and further challenge the training in the next level

### Theme 1. Need to meet exercise expectations with abilities

The first theme highlighted the importance of providing participants with an appropriate level of challenges and tailored modifications to maintain positive attitudes toward exercise. Although many appreciated the program's preset progression for fostering confidence and achievement, others found irrelevant content led to boredom and disengagement. Two subthemes were identified, with illustrative quotes presented in text box 2.

Text Box 2 **Representative quotes for Theme 1**

**Table:** 

Participant 3 stated, “I would've liked to have had the option to maybe try a more difficult level. And if I couldn't do it, then I couldn't do it. But at least I could try because I felt like at a certain point [*moving toward 8-9 weeks of the program*] I wasn't getting anything out of it. I wanted to see if I could do something a little bit tougher and you don't have that option. You're locked into your level.” [Low-responder] Participant 9 stated, “When I started I was confident and as they got slightly harder I got less confident if that makes sense. There was a lot of balance and that is my weak spot so I think I started to feel less confident the more it pushed you to do more. …. More anxious, that's the word.” [Low-responder]

Alt-text: Unlabelled box

#### Nonchallenging exercises caused boredom or lack of interest

Participants reported that too easy or slow exercises were unengaging and difficult to perceive as “exercise.” Some participants reported that the exercise intensity was insufficient, especially toward the end of the program, leading to a sense of disinterest and diminishing perceived benefits. Participant 3 shared these concerns.

#### Overly challenging exercises created feelings of frustration, anxiety, and loss of confidence during performance

In contrast, when the exercises were perceived as too difficult, participants reported feelings of frustration, anxiety, and decline in confidence. Some participants skipped the movements entirely, whereas others did their best to keep up (eg, pause/start the video repeatedly, use a chair for balance support). Participants commented that their initial fitness and balance levels made certain movements difficult to perform, which led to a loss of confidence over time. The loss of confidence. Participant 9 expressed these concerns.

### Theme 2. Preference for personalized content and tools to support exercise behavior change

The second theme identified the importance of providing contents and behavioral modification tools that are matched with participants’ interests, goals, and needs. Three subthemes were identified, with illustrative quotes presented in text box 3.

Text Box 3 **Representative quotes for Theme 2**

**Table:** 

Participant 8 remarked, “The automated calls I liked at first but then when I was exceeding the number of times of doing the exercises, it wouldn't allow me to input that information, so it was like okay then nobody really cares that I did it five times this week, they just wanted to make sure I did it two or three.” [Responder] Participant 11 commented, “Maybe if you add the article at the end of the video as a cool down period, it might get more attention. The lady can say, ‘As you cool down, remember or think about this’ and then they could throw that slide up for that article that they want to—that would relate to that week's workout.” [Low-responder] Participant 6 stated, “I can take ownership and say that I could've done better at reminding myself. But maybe just a reminder call to say, ‘Hey, you know, don't forget to exercise this week’ probably would've been nice.” [Responder] Participant 12 noted, “…a progress report kind of thing on TEAMS app maybe, showing you what week you're on and what you've done in the past maybe could help motivate someone who is not feeling very well that week.” [Responder]

Alt-text: Unlabelled box

#### General, irrelevant content created feelings of disinterest and disconnection

Participants reported that articles contained important information about MS. However, many participants believed that the content was too general and irrelevant to themselves, which created feelings of disinterest. Participants expressed a preference for more personalized content focused on topics such as individual barriers, exercise, and cognition. Similarly, participants believed disconnection when the questions, response options, and feedback generated by the IVR system did not align with participants’ situation, goal, or achievement. The feeling of frustration was shared by participant 8 when the system did not allow the person to report accurate numbers of exercise bouts.

#### Lack of awareness of content

Many participants reported that they were unaware of the articles being included in the exercise app. To increase visibility, participants suggested various strategies, such as reminder text or notification within the exercise app. One specific suggestion to enhance its use was to include a reminder at the end of the exercise video, as noted by participant 11.

#### Participants wanted multiple communication channels to facilitate exercise behavior modification

Regarding IVR system, participants reported that the weekly calls provided reminders and positive reinforcement, yet, some participants expressed desire for additional prompts, such as email or text reminders sent before the scheduled exercise session, as participant 6 stated.

In addition, participants indicated a preference for multiple ways to report and track their progress throughout the program. Participants commented that the IVR system was not convenient for logging their performance or reviewing their entries. They proposed using the exercise app, emails, or callbacks to the IVR system for reporting. To stay motivated, participants wanted to track their progress visually through graphs, summary reports, or rewards sent via the app or email. Participant 12 noted that having these progress monitoring tools could enhance motivation.

### Theme 3. Importance of accountability and human interaction to increase participation

The third theme identified the importance of optimal human interaction and support to increase participation through a sense of accountability. Two subthemes were identified, with illustrative quotes presented in text box 4.

Text Box 4 **Representative quotes for Theme 3**

**Table:** 

Participant 11 remarked, “I know it would be a lot of phone calls, but week 3, this group could be called, and week 2, this week could be called. … if I would have been asked so many weeks out, ‘Is it challenging enough?’ [*I could say*] ‘Well, I'm still doing that–I'm still doing the exercises, but could I have a little bit more?’ And that person could say, ‘Okay, let's look at what you're doing. You're in Group B. Group A is a little bit more challenging, would you be interested in that?’ and then see if they could.” [Low-responder] Participant 21 said, “To have somebody or group, you know, that is going through the exercise that, you know, working on going through the exercise together, that would probably actually be helpful, as well, to be able to motivate each other.” [Low-responder]

Alt-text: Unlabelled box

#### Human connection enhances feelings of support

Participants preferred occasional check-up calls with research staff, ideally every 3 to 4 weeks, to supplement or replace the IVR system. These calls can potentially resolve technology issues (eg, inability to open videos/articles, IVR errors, and loss of ID/password) and answer exercise-related questions (eg, exercise adaptations, difficulty, and clarification of exercise movements). Participants reported that 1-way IVR communication often led to frustration, as participants did not have options to call back or provide detailed responses beyond simple yes, no, or number. The value of human support was discussed by participant 11.

#### Exercise companionship enhances motivation

Participants reported that having an exercise companion enhanced their motivation to participate. Many participants relied on a companion (spouse, children, grandchildren, and members in a support group) for physical assistance and encouragement. The support could be done in-person or through videoconference, as noted by participant 21.

### Theme 4. Perceived barriers to program participation

The fourth theme identified several factors that led participants to lapses in participation. Three subthemes were identified, with illustrative quotes presented in text box 5.

Text Box 5 **Representative quotes for Theme 4**

**Table:** 

Participant 1 remarked, “My MS is super affected in the summertime. So, during the summer months it was—oh my gosh—hit or miss. Maybe once a week I was doing something successful. But even on a week-to-week basis there would be times where I wouldn't do the exercises at all because literally all I could do was get out of my bed, and like get to my couch in the living room.” [Responder] Participant 18 pointed out, “when I get off work after being there ten hours—ten hours is the shift I work now—so, I know that exercise is supposed to give you more energy, but I sometimes shoot myself in the foot when I'm fatigued, instead of going to exercise to get more energy.” [Low-responder] Participant 17 said, “I found out that I had a bulging disk that I didn't know I had. So, we may have to pause you on doing the end of it and I think at that point may have had four or five more weeks left to do. I don't even think I had that much left to do. I was really close to the end but I couldn't finish it out.” [Low-responder]

Alt-text: Unlabelled box

#### Daily variation of MS symptoms

MS symptoms, such as fatigue, heat sensitivity, and spasticity, varied daily or seasonally and were reported as barriers. When the symptoms were severe, participants avoided exercise to prevent exacerbating their condition. Participant 1 shared the challenge of managing symptoms in the summer.

#### Life stressors and responsibilities

Participants often prioritized work, caregiving, and other responsibilities over exercise. The fatigue after long hours of work was shared as a barrier to exercise by participant 18.

#### Unexpected injury or health issues

Participants reported unexpected health issues and nonstudy-related injuries during the intervention, such as MS relapses, broken bones, and rotator cuff injuries. These incidents prevented participation in the program, as noted by participant 17.

### Theme 5. Resources to reengage participants in continued exercise

The fifth theme revealed that participants continued the program throughout the follow-up period because of the perceived benefits of the intervention. The exercise videos were particularly valued as good resources for maintaining exercise. However, participants suggested that additional supplementary materials could have further supported re-engagement. Two subthemes were identified, with illustrative quotes presented in text box 6.

Text Box 6 **Representative quotes for Theme 5**

**Table:** 

Participant 16 said, “When I first started the program, I was walking anywhere from 15 to 20, 25 minutes a day. I got to do that five times a week taking my breaks and walking. And now, it's almost... I can walk a while. … That program, it changes everything; not just physically, but it makes you change how you schedule, and you notice that you improve outside of yoga—through the program—your mental space, everything is better because you're taking that time—that yoga was part of me taking time for me.” [Low-responder] Participant 11 commented, “After Week 12, it stopped as far as new stuff. And I—for repetitive stuff, it became very mundane. So, in my opinion, if the study could have said, ‘Well, week 12 is what we're aiming at getting people; but if there's anybody that would be interested in extending past that week 12?’ Then that's what I would have liked is more combination of exercise that would extend me past the week 12. … If we completed it in B and the therapist signed off and said, ‘Yeah, you're doing everything great; it looks like you've improved your flexibility. Do you want to go to the next level?’” [Low-responder]

Alt-text: Unlabelled box

#### Perceived benefits motivate to continue the exercise

Participants reported improvements in flexibility, balance, coordination, and reduced muscle stiffness. Also, participants reported increased energy, which made daily activities, such as shopping and walking, easier. Participants noted that the program helped alleviate MS symptoms, including better sleep and less pain. In addition, participants reported that exercise was used as a coping mechanism for life stressors, such as parenting or family loss, by providing stress relief and emotional control. These benefits across physical and psychosocial aspects were shared by participant 16.

#### Postintervention resources as a reminder

Some participants suggested ways to increase motivation and exercise volume during the follow-up period. These include supplementary materials such as videos with fewer instructions or new challenging movements, as along with as a summary sheet of exercises as a reminder. Together, these resources could provide quick access to the exercises, addressing feelings of boredom and disinterest toward the end of the program, as shared by participant 11.

## Discussion

This qualitative study investigated differences in the experiences, perceptions, and needs of participants with MS regarding the content and implementation procedures of a 3-month tele-exercise intervention based on their success in increasing PA levels. Based on the findings, we identified key areas for improving the design and delivery of the intervention, as well as the postintervention period, with the goal of developing an optimal tele-exercise intervention to enhance participation and engagement for people with MS.

The lack of notable group differences may stem from shared characteristics among participants, such as similar disabilities, physical symptoms, and common barriers to program participation. In related research, studies often compare distinct participant groups (eg, health care providers vs people with disabilities, health care providers with different professions)^37^^,^^38^ to examine differences in experiences and perceptions. The absence of group differences could also be attributed to the classification method, which used a “snapshot” of PA before and after the intervention, as well as potential self-report bias using the GLTEQ to estimate PA in pragmatic settings. Furthermore, because all participants were interviewed after completing the 1-year TEAMS study, ranging from 371 to 540 days from the baseline assessment date, this time gap may have influenced their responses, potentially explaining the discordance between the qualitative feedback and responder status.

Many participants in both groups noted experiencing mismatches between their exercise challenges and program's preset levels, particularly as the intervention moved forward (Theme 1). Although the TEAMS study included various accommodations (eg, 6 levels of preset modifications, 3 variations for each level, and tailored the level based on an onsite, clinician-led Timed 25-Foot Walk Test), participants highlighted the need for additional adjustments over time because of nonlinear progress and sudden changes in health status (Theme 4). Our findings reaffirm the need of building flexible, individually tailored programs for this diverse population of people with MS.

The modifiable factors identified in this study support the development of an adaptive intervention, which maintaining scientific rigor while accommodating individual needs. In this design, participants begin with a primary intervention, and their response determines whether they continue (responders) or transition to a secondary intervention (low-responders). This design operationalizes how, when, and based on which measure to alter treatment, ensuring systematic allocation to the most appropriate treatment.^23^ Although widely used in health promotion interventions for weight management,^39^, ^40^, ^41^ drug abuse,^42^^,^^43^ and depression,^44^^,^^45^ adaptive intervention design has yet to be applied to MS-specific programs.

Participants suggested conducting needs assessments every 3 weeks via phone or video conference call with research staff (Theme 3). This aligns with findings from weight loss studies that recommend periodic checkpoints to adjust interventions (ie, sessions 3 and 7).^46^, ^47^, ^48^, ^49^ Implementing regular human support checkpoints would provide the assistance participants desire, allowing them to ask questions about the exercise program and report any health changes that could temporarily hinder participation (eg, MS relapse, worsened symptoms, or injury) (Theme 4).

Intervention responses can be measured by PA level or adherence rate, such as minutes spent watching the exercise videos. Based on a predetermined threshold, responders can continue the asynchronous exercise training, as was the case for participants in this study, whereas nonresponders may benefit from synchronous programs with additional support. In this study, a similar number of participants in both groups valued group exercise with a coach, research staff, or peer (70% for responders [n=3/10] vs 75% for nonresponders [n=9/12]) for accountability and motivation, reflecting previous research emphasizing the importance of human connection in MS interventions.^17^^,^^18^^,^^20^, ^21^, ^22^

Participants appreciated behavioral change tools, such as educational articles and the IVR system, but highlighted a need for improvement (Theme 2). Preferences included participants indicated personalized reporting and monitoring options, such as apps, email, or callbacks to the IVR system, to better support self-regulation and progress tracking. This finding is consistent with previous research demonstrating the value of self-monitoring in promoting PA behavior among people with MS.^50^

Given the likelihood of health challenges in long-term studies, initial assessments alone are insufficient to determine a participant's 3-month exercise regimen. Adjustments should be made at various timepoints throughout the intervention to address individual characteristics, such as functional ability and preferences, as well as the appropriate level of human support. Future studies should explore tailored tele-exercise programs to optimize engagement and outcomes.

### Study limitations

This study had limitations. First, participants were primarily from urban areas with relatively stable internet, limiting the applicability of findings to rural populations with poor connectivity. Second, conducted during the coronavirus disease 2019 lockdown, responses may also reflect pandemic-specific experiences. Third, participants may have been more motivated to engage in exercise than the general MS population, as all had volunteered for the TEAMS study.

## Conclusions

This study identified modifiable factors that could enhance tele-exercise programs for people with MS, emphasizing the need for personalized, ongoing content modifications, optimized human interaction, and postintervention resources. These findings offer valuable insights for developing tailored interventions to sustain participation and maximize long-term benefits.

## Disclosure

T.M. received consulting fees from the American Physical Therapy Association, New Balance Foundation Obesity Prevention Center, The Obesity Journal, PLoS One, Novo Nordisk, and Heart Rhythm Clinical Research Solutions. The other authors have nothing to disclose.
